# Primary Prevention of Food Allergy—Environmental Protection beyond Diet

**DOI:** 10.3390/nu13062025

**Published:** 2021-06-12

**Authors:** Hanna Sikorska-Szaflik, Barbara Sozańska

**Affiliations:** Department and Clinic of Paediatrics, Allergology and Cardiology, Wroclaw Medical University, ul. Chałubińskiego 2a, 50-368 Wrocław, Poland; bsoz@go2.pl

**Keywords:** allergy, food allergy, environmental factors, primary prevention

## Abstract

A food allergy is a potentially life-threatening disease with a genetic and environmental background. As its prevalence has increased significantly in recent years, the need for its effective prevention has been emphasized. The role of diet modifications and nutrients in food allergy reduction has been extensively studied. Much less is known about the role of other environmental factors, which can influence the incidence of this disease. Changes in neonates gut microbiome by delivery mode, animal contact, inhalant allergens, oral and then cutaneous allergen exposure, air pollution, smoking, infections and vaccinations can be the potential modifiers of food allergy development. There is some data about their role as the risk or preventive factors, but yet the results are not entirely consistent. In this paper we present the current knowledge about their possible role in primary prevention of food allergies. We discuss the mechanisms of action, difficulties in designing accurate studies about food allergy and the potential biases in interpreting the connection between environmental factors and food allergy prevention. A better understanding of the role of environmental factors in food allergies development may help in implementing practical solutions for food allergy primary prevention in the future.

## 1. Introduction

Food allergy, a potentially life-threatening condition, is defined as an adverse reaction occurring reproducibly on exposure to a specific food. It can be IgE mediated (an immediate onset of symptoms caused by mediator release induced by IgE binding antibodies, mast cells and basophils), non-IgE mediated (delayed onset of symptoms connected with T-cell inflammatory responses) and also mixed IgE and non-IgE mediated food allergy [1].

Food allergies are diagnosed in up to 10% of the population (mostly children) and the prevalence has been increasing in the last decades [2]. The disease may affect children’s growth, patients’ quality of life and can be potentially life-threatening, even after exposure to a very small amount of allergen. The gold standard for food allergy diagnosis is the double-blind, placebo-controlled food challenge (DBPCFC) [3]. Despite significant progress in understanding food allergy epidemiology and involved mechanisms, treatment remains mainly based on exclusion of the harmful food from the diet. There are also many questions to be solved about food allergy pathogenesis. Genetic predisposition, epigenetic modifications and environmental exposures may be the risk factors. However, the possibility of some environmental interventions seems to be an opportunity for its primary prevention.

The influence of diet and nutrients on food allergy risk has been extensively studied. Early introduction of peanuts in the diet of high-risk infants reduced the peanut allergy rate in the following years [4]. Unfortunately, similar intervention with other food types gave less certain results [5,6]. The role of vitamin D, antioxidants, vitamins, pre- and probiotics and other diet ingredients were also extensively studied [7]. In the ‘dual-allergen exposure’ hypothesis the role of cutaneous contact with allergens rather than oral early in life was proposed to increase the harmful effect [8]. Much less is known other than dietary environmental factors that affect the risk of food allergy and may potentially be the strategic tool for its avoidance [9]. Modification of microbiome by delivery mode, inhalant allergens exposure, air pollution, smoking, infections and vaccinations may influence the immunological system and its reactivity to different allergens. It is known that these factors can act on different ages (pregnancy, infancy, childhood, adulthood) and modify the risk of allergic diseases. What is their role in food allergies?

**Figure 1:**
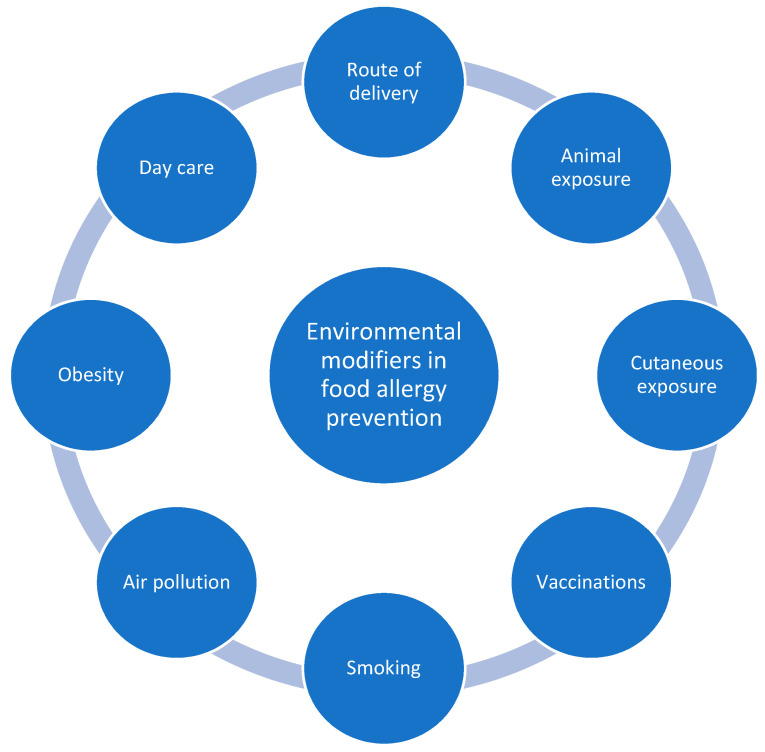
Primary prevention of food allergy—potential environmental modifiers.

In this review we discuss the possibilities of primary prevention of food allergies given by environmental factors other than dietary and nutrients (Figure 1). We present the newest research on potential mechanisms of action and perspectives of implementing their findings in the practical reduction of food allergy risk.

## 2. Route of Delivery

The role of the gut microbiota in many processes of the human organism has been thoroughly investigated. Microbiota is believed to influence the development and maturation of the intestinal lymphoid tissue, strengthen and maintain the integrity of the mucosa and activate the intestinal immune defense [10]. Studies published recently suggest an important role of the intestinal flora in the development of food allergy. The disruption of the original microbiota configuration would have been related to higher risk of this disease [11,12]. Gut microbiota is supposed to have a regulatory role in the manifestation of food allergy but the exact mechanism of this influence is still under investigation. Presumably, it influences the immune system by altering the host’s metabolism and altering adaptive immunity [13]. Caesarean section is among the factors potentially responsible for dysbiosis, defined as a change in the microbiota composition, function and imbalance of the gut microbiota homeostasis. Therefore, caesarean section is being considered as having the potential to increase the risk of food allergy [9,14]. Infants who are born through the birth canal are directly exposed to their maternal gastrointestinal flora by mouth, whereas caesarean born infants do not have such a high direct exposure, allowing lower levels of colonization from maternal skin microbiota and areas of low biomass to colonize the neonatal gastrointestinal tract. Compared to infants born naturally, babies born by caesarean section have lower levels of Bacteroides, E-shigella and E. coli [15] and higher levels of Clostridium difficile [16] in the gut microbiota. However, Dominguez-Bello et al. showed that the microbiota of infants’ skin and mouth acquired a more adult-like configuration after the first week of life and this was independent from the delivery mode. As the difference in microbiota composition is relatively short-lived, considering its influence on food allergy development should be explored cautiously [17]. Omission of the maternal flora is suspected of predisposing to a proallergic Th2 phenotype and to food allergy simultaneously [17]. These observations are in line with the biodiversity hypothesis assumption that microbial exposure in early life regulates the immune response and has a role in the prevention of allergic diseases [18].

A Swedish longitudinal cohort study conducted on more than one million children reported an increased risk of food allergy in children born by means of elective or emergency caesarean delivery compared to those delivered vaginally. Moreover, infants born large for gestational age or with low Apgar score also had higher risk of developing food allergy. In contrast, children born <32 week of pregnancy were less likely to be diagnosed with food allergy in adolescence [19]. In another study, caesarean delivery was connected to an increased risk of cow’s milk allergy in children, regardless of whether it was an elective or emergency caesarean section [20]. In the population-based birth cohort, the Pollution and Asthma Risk: an Infant Study (PARIS) sensitization to food allergens also tended to be more frequent in children born by caesarean section [21]. Increased risk of food allergy, but only in predisposed children with a family history of allergies, was shown by Eggesbø et al. Children whose mothers were allergic and who were delivered by means of caesarean section had a seven-fold increased risk of parentally perceived reactions to egg, fish or nuts and a four-fold increased risk of confirmed egg allergy. In contrast, children with no family history of allergy born by caesarean section did not show a significantly increased risk of food allergy [22]. Similarly, in a Greek cohort study it was shown that caesarean section predisposed food allergy development and that risk was more pronounced in children with allergic family history [23].

In the study of Norwegian children, 6.8% of the study group developed symptoms of food allergy during the first two years of life. In this group of food allergic children, 7.6% were delivered by caesarean section and 6.5% were born vaginally. As the difference was not significant, the authors stated that there was no increased risk of food allergy in the first two years of life in children born by means of caesarean section compared to those delivered vaginally [24]. In the Urban Environment and Childhood Asthma birth cohort study, the children were followed through the age of five years and the influence of many factors on food allergy to milk, egg and peanut were checked. Type of delivery appeared to have no effect on the occurrence of food sensitization or food allergy in children [25]. Pyrhönen et al. did not find an association between the mode of delivery and allergy manifestation, including food allergy during the first four years of life. However, the authors admit that due to the wide confidence intervals in their study, it is not possible to completely exclude the existence of a relationship between caesarean section and the development of allergic symptoms in children [26].

The discrepancies in the studies’ results may be partially explained by the differences in the study design and enrolled populations (for example, different family histories), methods of diagnosis and time of observations. Despite the fact that DBPCFC is widely considered as the "gold standard" for the diagnosis of food allergy [2], the diagnostic procedures varied among studies. Assessing specific immunoglobulin E (sIgE) or conducting skin prick tests were used, but sometimes the lack of an objective way to measure the outcomes was applied. The cutoff points when food allergy was diagnosed, or variability in the food allergen chosen in the diagnostic process, can also be a shortcoming and can make it difficult to compare different study results. Some studies are also vulnerable to bias due to missing data from study participants or from conducting the trial using specific groups of a population. Women giving birth by cesarean section have easy access to health care providers, so in the case of allergy symptoms in their child they found a specialist consultation quickly. On the contrary, people with minimal contact with medical care may not seek advice from an allergy specialist or some symptoms may be simply ignored. Interestingly, in the EAT study where the DBPCFC was used to diagnose food allergy, despite the differences in the composition of infants’ gut microbiota, no difference in the risk of food allergy was found in children [27].

Considering that most of the studies on the relationship between caesarean section and food allergy have not reach statistical significance, it is not possible to unequivocally state the influence of this type of delivery on the occurrence of food allergies in children. However, as the percentage of caesarean sections (including those based on maternal request) is increasing nowadays, further studies should be conducted to establish the role of caesarean section in food allergy development.

## 3. Animal Exposure

Another factor conceivably related to the onset of food allergy is animal exposure. Available studies suggest that contact with animals, both pets and farm animals, has a possible influence on the occurrence of atopic disease. This is based on the concept, similar in assumptions to the aforementioned biodiversity hypothesis, that early exposure to animal allergens reduces the organism’s susceptibility to allergic diseases. Keeping a pet increases exposure to endotoxin and might therefore contribute to a lower risk of atopy [28]. Most studies focus on the effect of exposure to animals on the development of atopy, asthma and allergic rhinitis. Numerous studies confirmed the lower risk of atopy in children living on the farm [29,30,31]. However, the rural environmental protection may be connected not only with farm animal contact but also with some dietary habits, like drinking unpasteurized milk [32].

The role of exposure to pets and food allergies and other allergic diseases has been studied but the results are conflicting [33,34]. Regarding the interventional Enquiring About Tolerance (EAT) study participants, Marrs et al. showed that owning a dog is a potential protective factor against food allergy in children. Researchers enrolled three-months old infants and then examined the children at one and three years of age, establishing if the allergy to egg, peanut, milk, wheat, cod and sesame developed in connection to pet ownership. Having a dog appeared to reduce the odds for food allergy by 90%. Moreover, the protective effect increased with the number of dogs owned by the child. Keeping a cat had an effect on reducing the risk of food allergy only if a dog was also present in the household [35]. In the HealthNuts study population, the relationship between pet ownership and egg allergy was studied. Keeping a pet appeared to be a protective factor on egg allergy also in children without a family history of allergic disease [36].

On the contrary, in a study conducted in the United Kingdom, no association between pets at home and the development of food allergy in children up to two years of age was shown [37]. Levin et al. revealed that exposure to farm animals was related to a decrease of food sensitization and food allergy. The authors underlined the difference between urban and rural environments regarding risk and protective factors of food allergy [38].

A protective effect of animal exposure on developing food allergy has been found in some studies, whereas others do not show this association. Distinguishing children with positive allergy family history from those without is important when final conclusions are made about the pet effects on allergy. The potential role of animal exposure in prevention of food allergy is still inconclusive. The inaccuracy may be a result of the variety of study designs. Some analyzed infants, while others focused on older children. Furthermore, the diagnosis of food allergy in the studies was made on the basis of diverse methodology. The gold standard to diagnose food allergy is DBPCFC [2], but this was used in just a few studies, such as in the EAT study [35]. In many studies, the diagnosis was based on parental reports or specific IgE testing or skin prick testing, which may lead to overdiagnosis of the disease. Not using validated gold standard diagnosis is a weakness of these studies and makes the results unreliable and not comparable with the results of other well-designed surveys.

What is more, distinguishing children with positive allergy family history from those without is important when final conclusions are made about the pet effects on allergy. The potential role of animal exposure in prevention of food allergy is still inconclusive.

## 4. Cutaneous Exposure—Dual-Allergen Exposure Hypothesis

The dual-allergen exposure hypothesis proposed by Lack suggests that low-dose cutaneous exposure to food allergens is a risk factor of food allergy, while early intake of an allergen induces oral tolerance [8]. Du Toit stated that this theory is a precise illustration of one of the mechanisms of developing a food allergy [39]. When the skin barrier is impaired, such as in atopic dermatitis, this skin exposure might be even greater, which may also partly explain the frequent coexistence of atopic dermatitis and food allergy. In a systematic review, a strong association between atopic dermatitis, food sensitization and food allergy was found. In most cases atopic dermatitis arises before the development of food sensitization, which supports the theory of a causal relationship [40]. In the aforementioned population of the HealthNuts study, Martin et al. showed that atopic dermatitis in infancy, especially with early and severe onset, is a strong risk factor for IgE-mediated food allergy, and that eczema increases the odds of food allergy nearly five times compared to children with healthy skin [41]. Similar risk factors of food allergy—early onset of atopic dermatitis and its severity—were shown in children at three months of age [42]. Studies demonstrated the risk of sensitization to peanuts or peanut allergy after skin exposure to peanut allergen in household dust [43]. To determine the route by which infants become sensitized, the authors observed peanut protein levels in the child’s environment, household peanut consumption and the development of peanut allergy in children. They made a conclusion that infant’s environmental exposure to peanut antigens in the dust through an impaired skin barrier is a probable route for peanut sensitization and allergy [43]. Another study confirmed the increased risk of peanut food allergy after environmental exposure in infancy but also stated that consumption of peanuts in these infants in the first year of life was a protective factor against peanut allergy [44]. Peanut proteins were also detectable in the house dust of families that restricted peanuts and peanut products at home [45]. Taking into consideration environmental exposure of other food allergens, Trendelenburg et al. found that hen’s egg allergen was also detectable in house dust samples. They concluded that high environmental exposure to hen’s egg allergens may also be a risk factor for cutaneous sensitization, especially in small children with atopic dermatitis [46]. As a defect in the skin barrier is proposed to be a risk factor of development of peanut allergy, attention is also paid to the filaggrin-filament binding protein in the stratum corneum of the skin. Filaggrin mutations lead to increased skin permeability and reduced skin barrier function [47]. To investigate the association between filaggrin loss-of-function mutations and peanut allergy, a case-control study was conducted and showed a strong and significant association with peanut allergy in the food challenge-positive patients [48].

The dual-allergen exposure hypothesis suggests that somehow if the skin barrier can be improved then the risk of food allergy can be reduced. Proper treatment of atopic dermatitis may lower the risk of food allergy. What is more, actions taken to reduce the risk of eczema may lead to decreasing the food allergy occurrence, as was shown in case of reducing bathing frequency and protection against eczema in infants [49]. Therefore, for primary prevention of food allergy the appropriate skin care should also be recommended. However, as Perkin et al. found, moisturizing infants’ skin can also promote food allergy. The authors explained that moisturizers can make it easier for food allergens to break the skin barrier or damage the skin barrier and enable the penetration of the food allergen [50]. Beyond proper skin care, a second important aspect of dual-allergen exposure theory is the importance of introducing foods orally at the right time to stimulate tolerance. As it is stated, a window of opportunity exists during the child’s first year of life within which there is a possibility to influence a tolerogenic response [39].

## 5. Vaccinations

As a Bacillus Calmette–Guerin (BCG) vaccination has the potential to stimulate Th1 action involving cytokine response patterns and simultaneously inhibiting the Th2 immunologic response [51], the association between vaccination and the risk of atopic disorders has been studied. In the review by Arnoldussen et al., two original studies considering the association between BCG vaccination and a risk of food allergy were discussed [52]. The results of these studies were too heterogeneous to be pooled as they did not show statistically significant evidence that BCG vaccination has the potential to reduce the risk of food allergy or food sensitization [53,54]. However, in one of these studies Steenhuis et al. presumed that there could be a smaller beneficial effect of BCG vaccination, as in vaccinated children less eczema and significantly less use of medication for eczema was shown. The authors paid attention to the timing of when children got a vaccine. It is likely that BCG vaccination may have a protective immunomodulatory effect when it is administered in the early neonatal period, but not later [54]. The effect of BCG on the risk of atopic diseases, time of vaccine administration and also the impact of neonatal vitamin A supplementation were aims of the study conducted by Kiraly [55]. There is a possibility of BCG vaccination influencing food allergy occurrence in children under certain conditions, however, the results of the study are inconclusive [55].

There is little evidence that BCG vaccination may have a potential to reduce a risk of food allergy. More studies are needed to verify the association between atopic sensitization and administration of BCG. Therefore the latest guidelines of the European Academy of Allergy and Clinical Immunology (EAACI) are against recognizing the BCG vaccination as a method of food allergies prevention [56]. Pertussis vaccine was also considered as having a positive effect on reducing the risk of allergies, but Venter et al. found no such association in investigated children [57].

It is worth underlining that the studies conducted on large groups of patients showed no increased risk of atopic diseases including food allergy, confirming the safety of vaccinations [58,59]. Parents concerned about the possibility of developing atopic diseases in their children should be reassured about the safety of vaccines given early in life.

## 6. Smoking

Exposure to tobacco smoke is another factor possibly contributing to the development of food allergy [60]. The mechanism of action is still unclear, but there are some assumptions. It is possible that breathing air polluted by tobacco smoke in the first months of life may disrupt the skin barrier and lead to exposition of food allergens via this way. As it was described earlier in this article, skin exposition to food allergens may promote food allergy [6]. Moreover, cigarette smoke reduces the activity of Th1 lymphocytes and thus may contribute to the development of allergic diseases [61].

The role of exposure to tobacco smoke in utero and postnatally for IgE sensitization to allergens in children at four years of age was studied. The results were different depending on the time of exposure. Infants exposed to smoke only during pregnancy did not present a higher risk of sensitization to food (cow’s milk, hen’s egg, peanut, soy, wheat, cod fish) and indoor inhalant allergens. However, small children whose parents were smoking during the first months of the children’s life demonstrated higher risk of sensitization and the effect was dose-dependent [62]. While examining the same group of patients, however, conducting a longer follow-up of children up to 16 years of age, Feldman stated that exposure to tobacco smoke in the second month of life increased the risk of food allergy. It remained significant for egg and peanuts (OR 1.79 and 1.50, respectively) [63]. Similar results were presented in a meta-analysis of studies—passive smoking during childhood but not in utero was associated with an increased risk for food allergy [64].

In contrast to the results mentioned above, a population-based study conducted in Sweden revealed a nonsignificant relation between exposure to tobacco smoke and allergies in children. Only children exposed to secondhand smoke and simultaneously having a family history of atopic diseases showed an increased risk of developing an allergy. Researchers emphasized the synergistic effect of inheritance and the influence of passive tobacco smoke exposure [65].

Smoking is a known risk factor for the development of respiratory tract diseases in children, and also allergic diseases. Of course, research on the influence of smoking on the occurrence of allergic diseases has its limitations—the effect of accompanying factors cannot be ruled out. Moreover, these studies are often based on data collected from an interview the parent does not always want, or they do not always remember factual information. Nevertheless, considering that almost every second child is exposed to tobacco smoke [66] and about 14% of children are exposed to smoking during pregnancy [67], educational information about the harmfulness of smoking and the effects of passive smoking on children should be an important part of medical appointments.

## 7. Air Pollution

Poor air quality indoors may result not only from exposure to tobacco smoke but also to the quality of the outside air. Traffic-related pollutants, including particles and nitrogen oxides (NOx), are said to induce inflammation of the airway and may increase airway responsiveness [68]. Air pollution is also a possible environmental factor playing a role in food allergy development. Diesel exhaust particles may act as adjuvants to allergen and in that way escalate the sensitization response. What is more, NOx are associated with suppressing the Th1 response and promoting the Th2 proallergic response [69]. The effect of gene–environment interactions in explaining the impact of air pollution on allergic diseases has been proposed. Melén et al. studied the impact of air pollution on a single nucleotide polymorphism. They revealed that variants in the glutathione S-transferase P1 and tumor necrosis factor (TNF) genes modify the result of early long-term exposure to air regarding the sensitization to allergens in children [70]. Canadian children who participated in a prospective longitudinal national birth cohort were observed for over one year and NO2 concentrations in the place of their location were measured. Then the occurrence of allergy to inhalant and food (milk, eggs, peanuts, soy) allergens was determined. The authors showed that exposure to air pollution during the first year of life was correlated with positive results of skin prick tests with the mentioned food allergens at the age of one year. Similar to tobacco smoke studies, the allergic effect was not seen when the exposure to traffic took place during pregnancy [71]. Other prospective birth cohort studies analyzed the impact of air pollution in children from birth to four years of age [72]. The findings confirmed the positive, however nonsignificant, association between sensitization to food allergens and pollution exposure.

As the exposure to air of poor quality can be harmful for children’s health, it is important to prevent this exposure as much as possible. Limiting going outside on days when the air quality is very poor may even be needed. Actions leading to the improvement of air quality are also important—limiting car traffic where possible, using public transport and changing fuel to limit pollution.

## 8. Obesity

The incidence of both atopic diseases and obesity in the pediatric population is steadily increasing. Obesity can be described as a chronic systemic inflammation resulting from the interaction between adipocytes and macrophages recruited to adipose tissue in obesity. TNF-α, leptin and adiponectin are involved in this inflammation, and an increase in gene expression of proteins related to inflammation in obese people was shown—including genes of TNF-α, chemokines, IL8, MCP-1, complement proteins and other acute phase proteins [73,74]. This inflammatory state connected to obesity may be associated with an increased risk of atopic diseases, including food allergy. Another possibility is the influence of leptin—a hormone derived from adipose tissue. Its concentration is significantly increased in obese people [75]. It affects lymphocytes and the production of Th1-specific cytokines, and at the same time inhibits Treg proliferation, which may promote allergic diseases [76].

In a National Health and Nutrition Examination Survey, a relationship between obesity and allergic disease including food allergy was shown. Researchers examined children aged 2–19 years old and tested them for total IgE and specific IgE to i.e., peanut, milk, egg, and, in the case of six-year-old children or older, also to shrimp. It appeared that increased weight was associated with higher allergic predisposition and the effect was more pronounced in obese children compared to those who were overweight. However, the authors underline that this result cannot be understood unequivocally that obesity is a certain risk factor of food allergy. There is a possibility of the mutual influence of both these conditions, and perhaps there are additional issues influencing this relationship [77].

Irei et al. checked the association between overweight and food allergy in a population of 2027 children aged 9–13 years old from Japan, Taiwan and Vietnam. They showed inconsistent results in children from different regions—there was an association between being obese and having food allergy but only in part of the studied population [78]. However, even the authors admit that their study assumed only a questionnaire declaration of the presence of allergies, which may lead to a distortion of the result. Difficulties when interpreting the research results may include the different age of the respondents, methods of assessing the occurrence of food allergy (information from the parent/from the patient itself/objective sIgE assessment) and differentiated classification of BMI values as overweight and obesity. Therefore there is no strong evidence to consider obesity as a risk factor of food allergy.

## 9. Daycare

Increased risk of food allergy among children attending daycare facilities is also plausible. In Sweden, over 10,000 children were studied and revealed an association between daycare attendance and allergy symptoms (including allergic reactions to food). Children cared at daycare centers were more likely to present food allergy reactions compared with children cared for at home. It was more pronounced in children who started daycare before the age of one than in children who started attending daycare after the age of two [79]. In contrast, a recent study of 5517 participants aged 1 to 18 years did not show an increased risk of atopic diseases (including food allergy) in children attending daycare during the first year of life [80]. Similarly, Koplin et al. revealed lower risk of challenge-proven egg, sesame and peanut allergy in children at daycare in the first six months of life compared to those cared for at home [81]. This result may be related to the microbial exposure influence [9].

It could be assumed that attending a nursery or kindergarten could have a protective effect against allergies in a similar way as some other environmental factors—increasing contact with infectious agents would reduce the risk of allergies. However, since attending daycare does not have a clear antiallergic protective effect, the relationship is not unambiguously confirmed. It is also possible that the relationship between daycare and food allergy is not a causative nor protective one. It may be related to the fact that children at daycare centers are more often offered food that they would not get or try at home. What is more, they consume the meals because other children do. Sometimes this may lead to the occurrence of allergy symptoms. Possibly children taken care of at home who refuse some potential allergens somehow protect themselves against food allergy symptoms. Certainly, many factors have an influence, as a study of atopic wheezing showed that attending daycare can have a different effect (protective or not) depending on the varied expression of TLR2 genes in a child [82].

## 10. Conclusions

The knowledge about the role of environmental factors other than food in the development of food allergy is still surprisingly scarce. A better understanding of how external exposures interfere with the immunological system in food tolerance or food sensitization development is crucial for implementation of prevention methods. In the newest guidelines from the EAACI on food allergy prevention in infants and young children, none of the discussed possibilities reached a sufficient level of evidence to be recommended [55]. This may reflect the difficulties in designing accurate studies about food allergy; the dangers of elicitation bias, selection bias and losing participants to follow up; the importance of double-blind food challenges and the importance of ensuring representatives access across different populations. Therefore, there is an urgent need to conduct further studies organized on the basis of available guidelines for food allergy diagnosis and with objective measurements of environmental exposures.
